# Prognosis of Surgical Neonates

**Published:** 2012-01-01

**Authors:** Afzal Sheikh

**Affiliations:** Department of Pediatric surgery, The Children’s Hospital and the Institute of Child Health Lahore, Pakistan

The prognosis and outcome of surgical neonates travelled across many phases of development of modern sciences. In past the prognosis of surgical conditions, as whole regardless of the age, was poor. But with the advent of modern anesthesia and intensive care units and involvement of new diagnostic and therapeutic modalities the prognosis is now on the move. Contrarily, surgical neonates are a subclass of patients having wide differences in physiology, anatomy, diseases, immunity and response to the stress, as compared to older patients. Previously, dealt by the general surgeons, the prognosis and outcome of many surgical conditions of neonates were poor as many of them were considered mortal at that time. Even the congenital malformations that have excellent outcome today had poor outcome at that time. The outcome started improving with the development of pediatric surgery specialty that utilized new procedures, modify previous approaches and provided adequate postoperative care to pediatric patients and neonates. The outcome and prognosis varies disease to disease. Generally speaking many neonatal surgical disorders have now good outcome especially anorectal malformations, hirschsprung’s disease etc.

The development of pediatric surgery achieved many milestones and now many new specialties are being formed. Pediatric cardiac surgery, urology, orthopedic surgery, neurosurgery and so on are the examples. Similarly neonatal surgery should be considered as a separate domain as there is still a big room for the improvement of prognosis and outcome of surgical neonates.

The outcome and prognosis also depends upon various other factors which are called factors of poor prognosis. The prognosis can be judged at the admission of neonate with surgical condition. Manchanda et al presented a study in this issue of the journal to evaluate the prognostic factors of mortality in these patients. Many clinical and biochemical parameters were evaluated but gestational age, tachycardia, respiratory distress and mainly sepsis were proved significant on multivariate analysis however many other factors such as weight, temperature etc were significant on univariate analysis. We have also conducted a comparative study to identify the mortality in neonates of anorectal malformations where patients of anorectal malformations were divided in two groups one having congenital anomalies and other without anomalies. The mortality was statistically significant in the congenital anomaly group (P value less than 0.05). Although this was not subjected to univariate or multivariate analysis however associated congenital anomalies especially cardiac and major renal anomalies have additive effect over the mortality of these patients. Manchanda et al were not being able to construct a score for evaluation of the probable prognosis at admission; however, presence of these factors can alert the treating team about the critical condition of the neonate. More studies are welcome in this regard to construct a score for assessing prognosis of surgical neonates at admission.

## Footnotes

**Source of Support:** Nil

**Conflict of Interest:** None declared

